# Supplementary material for: Hierarchical structure and memory mechanisms in agreement attraction
Source: PLoS One. 2020 May 19;15(5):e0232163. doi: 10.1371/journal.pone.0232163 (PMC7237028; doi:10.1371/journal.pone.0232163)
Supplement: Attachment — Submitted filename: Response to reviewers.pdf [file pone.0232163.s001.pdf]

Response to reviewers

Reviewers' comments are in italics.

Reviewer #1

Low statistical power of both experiments:
Experiment 1: 43 participants for 16 conditions;
Experiment 2: 25 participants for 12 conditions;
This very small sample size is problematic even if the results do reach statistical significance (see Gelman and Carlin, 2014; Vasishth, Mertzen, Jäger, and Gelman, 2018)

With respect to Experiment 2, while the statistical properties of the response-signal technique unfortunately remain underinvestigated (see Liu & Smith, 2009), we were guided by the existing literature in determining our sample size; see for example:

- Van Dyke & McElree (2011): 20 participants for 8 conditions
- Xiang et al. (2014): 20 participants for 8 conditions
- McElree, Pylkkänen, Pickering & Traxler (2006): 26 participants for 6 conditions
- Göthe & Oberauer (2008): 24 participants for 24 conditions

The design is different than in a typical language experiment, because, at the individual level, much more data is collected per condition, since it is a multi-session experiment in which all experimental items are seen by all participants. All parameter estimates are made at the individual, and not group, level for statistical inference (we presented all individual participant data in Figure 3). If it would be helpful, we can follow the practice in many recent studies of including a table of parameter estimates on the individual level so readers can check the consistency of the parameter estimates for themselves.

The authors never clearly define their understanding of the term "attraction". In the introduction they write: "Attraction errors are characterized by the incorrect agreement of a target with an element that is not its grammatical controller". However, later in the paper, it seems that with the term attraction, they mean the effect of the attractor being plural compared to it being singular. This makes sense for the ungrammatical sentences, which have a singular target and a plural verb. Here it is common to use the term attraction to refer to a facilitation caused by the plural attractor. However, in the grammatical sentences, which have a singular target and a singular verb, it does not make any sense to me to call the facilitation caused by the intervening plural noun "attraction". In particular, within a content-addressable framework, what matters is the match with the retrieval cues, i.e., the verb's number. Hence, in the grammatical conditions, I would find it more straightforward to code the attraction effect in the opposite direction. This concern does not only relate to the presentation of the work, but --- even more importantly --- to the contrast coding of the statistical analyses and the validity of the conclusions concerning a content-addressable memory architecture that are drawn from the observed "attraction".

By 'attraction', we mean the *influence*, on agreement, from a number feature that is not on the subject head noun and that mismatches it. Indeed, the reviewer is right that the literature shows that this influence shows up in two opposite directions:

- It is detrimental in sentence production, but also in grammaticality judgments (whether the sentence is grammatical or not);
- It is beneficial in more passive tasks of sentence comprehension for ungrammatical sentences (giving rise to the so-called 'grammatical illusion'), as well as for grammatical sentences in some studies.

We now make it clear how the intricate pattern of evidence showing opposite results between production and comprehension can be interpreted at the end of the first section on theories of attraction:
"It is important to note that the cue-based retrieval mechanism that is assumed in studies of agreement in comprehension manifests differently from the mechanism assumed in agreement production. Indeed, whereas in comprehension studies the verb is presented with an agreement feature, such that this feature can provide a cue to subject retrieval, no such feature is present on the verb in production studies, such that other cues are assumed to be used for subject retrieval (being a NP, carrying nominative case, occupying a particular phrasal or linear position). Hence, whereas in sentence comprehension similarity-based interference manifests in terms of penalty due to *similarity in agreement features* of the subject and the attractor, no such penalty can manifest in sentence production: a subject retrieval error in production can only show up in sentences involving a feature mismatch between the subject and the attractor. As a result, while the same subject cue-based retrieval mechanism is argued to underlie agreement processing in production and comprehension, this mechanism shows up in terms of penalty due to agreement feature mismatch (of the two nouns) in production, but in terms of penalty due to number match (of the attractor and the verb) in comprehension (Villata & Franck, 2019)."

We also make this point clearer in the General discussion:
"Interestingly, although attraction in sentence production has traditionally not been interpreted as evidence for the involvement of a content-addressable memory system, a wide array of observations actually seem to attest to the role of the similarity between the agreement controller and the attractor. It is important to note that similarity effects in sentence production contrast with those reported in sentence comprehension in that they cannot show up in terms of agreement feature similarity, since agreement features are not available on the verb. Yet, they do manifest in terms of various morphological, semantic and syntactic features (Franck, 2017)."

In line with the existing literature, Experiment 1 using a grammaticality judgment showed penalty due to the presence of a feature mismatching the head, in both ungrammatical and grammatical sentences. The reviewer seems to have expected facilitation in grammatical sentences, as he writes that "in the grammatical sentences, which have a singular target and a singular verb, it does not make any sense to me to call the facilitation caused by the intervening plural noun "attraction" ". But this is not what the literature nor our results show: grammaticality judgment of both grammatical and ungrammatical sentences is penalized by the presence of a mismatching plural noun. Facilitation is only found with passive comprehension procedures. It therefore seems to us reasonable to keep the term 'attraction' to refer to the penalizing effect that both our experiments have shown, as well as the corresponding contrast coding which is the same for grammatical and ungrammatical

sentences. And we now make it clear in the section Overview of the study that attraction shows up in grammaticality judgments similarly to sentence production:

“Experiment 1 examines whether attraction arises in jabberwocky, and whether it shows sensitivity to structure as found in natural sentence production. We used a speeded grammaticality judgment task, which has been shown to consistently replicate attraction effects found in sentence production, with a significant penalty for sentences containing a mismatching plural attractor. Moreover, such a penalty is typically reported irrespectively of whether the sentence is grammatical or not (Franck et al., 2015, in French, and Haüssler & Bader, 2009, in German). The finding of a mismatch penalty in grammaticality judgments (which contrasts with the match penalty reported in comprehension studies using a more passive reading task) is due to the fact that, like in sentence production, participants cannot make use of the feature on the verb as a cue to subject retrieval, since the correctness of the verb feature is precisely what needs to be judged. A key property of grammaticality judgements is that this procedure has also been shown to replicate the syntactic modulation of attraction found in sentence production (Franck et al., 2015, Experiments 2 and 3).”

In the revised manuscript, we also now explicitly discuss the lack of difference we found between grammatical and ungrammatical sentences in the discussion of Experiment 1:

“Finally, attraction was found for both grammatical and ungrammatical sentences, and manifested as penalty due to number mismatch. Although this finding aligns with other studies that used a grammaticality judgment procedure in German (Haüssler & Bader, 2009) and in French (Franck et al., 2015, Experiment 3), it contrasts with findings from comprehension studies based on a reading task, in which the presence of a mismatching feature was found to facilitate the reading of ungrammatical sentences (Dillon et al., 2013; Tanner et al., 2014; Wagers et al., 2009, see Jäger, Engelmann & Vasishth, 2017, for a meta-analysis) as well as grammatical ones (Franck et al., 2015; Villata et al., 2018; Villata & Franck, 2019). In line with Franck et al. (2015), we suggest that the difference comes from how the two tasks draw on fundamentally different mechanisms. Attraction in reading for comprehension tasks, we propose, primarily reflects the process of subject identification in structure building: attraction is a side effect of identifying the subject, which takes advantage of the attractor’s mismatching feature due to the lower feature overlap between the verb and the attractor. In contrast, attraction in grammaticality judgment tasks taps into an explicit process of agreement checking for which the agreement features of the verb, which need to be checked, cannot be used as retrieval cues. In these tasks, misidentification of the attractor as the agreement controller can only lead to a detectable error when the attractor mismatches the controller, exactly like what is found in sentence production. Further research is necessary to fully understand task differences and their significant impact on sentence processing.”

Relatedly, the authors state in the Introduction: "When a subject NP that matches the verb is present, then the correct controller of agreement will fully match the cues and any attractor will only partially match it. Correspondingly, attraction in grammatical strings is expected to be weaker compared to attraction in ungrammatical strings. These predictions were borne out by computational simulations in ACT-R (Dillon et al., 2013)." Here, it does not become clear what they mean by "attraction". Only in the

Analysis section it becomes clear that attraction in grammatical sentences is coded as the effect of an attractor that MISMATCHES the verb (as compared to ungrammatical conditions, where it is the effect of an attractor that MATCHES the verb). In the context of ACT-R, such a coding is pretty uncommon, so the mentioned ACT-R predictions are very confusing. The authors should at least explicitly and clearly define already in the introduction what they mean by "attraction" in sentence comprehension in both ungrammatical and grammatical materials.

As we just made clear in our previous paragraph, grammaticality judgements always give rise to penalty in the presence of a mismatching feature, whether the sentence is grammatical or not. Nevertheless, we are thankful to the reviewer to have drawn our attention to the fact that one should not refer to 'attraction' when describing the facilitatory mismatch effect observed in more passive procedures of reading during sentence comprehension for grammatical sentences – indeed, one of our sentences made use of that term. We have now rewritten the corresponding paragraph accordingly:

"The hypothesis that cue-based retrieval is involved in the processing of agreement gained further support by studies of agreement in sentence comprehension. A number of studies using self-paced reading, eye-tracking and ERP methods have reported effects of a mismatching attractor taking the form of an illusion of grammaticality: in ungrammatical sentences, the presence of a plural attractor noun mismatching the singular head but agreeing with the verb decreases the perturbation normally found in ungrammatical sentences (Wagers, Lau & Phillips, 2009; Tucker & Wagers, 2010; Dillon et al., 2013; Tanner, Nicol & Brehm, 2014; Lago et al. 2015). Reports from the literature show that the influence of a mismatching plural attractor in grammatical sentences is generally absent, but when it is present, it also shows up in terms of faster reading times (Franck et al., 2015) or reduced comprehension errors (Villata et al., 2018; Villata & Franck, in press, and for similar evidence in children see Adani, 2010; 2014). Wagers et al. (2009) argued that the existence of an effect-size asymmetry between grammatical and ungrammatical sentences was actually expected under the hypothesis of a cue-based retrieval process in comprehension, supported by the same basic memory mechanisms as Badecker & Kuminiak (2007) proposed for production. According to this view, the agreeing verb in comprehension supplies retrieval cues to check for a controller NP in the parse. Such cues are expected to include information about the grammatical number of the candidate NP as well as its case or syntactic position. If the clause-mate subject NP does not match the verb in number, then no single NP will fully match the retrieval cues. But the presence of a plural attractor in the parse would partially match the cues, allowing the parser to (erroneously) satisfy the agreement requirement on some proportion of trials. When a subject NP that matches the verb is present, then the correct controller of agreement will fully match the cues and any attractor will only partially match it. Correspondingly, the effect of a mismatching number feature in grammatical strings is expected to be weaker compared to ungrammatical strings, and to also manifest in terms of processing facilitation. These predictions were borne out by both behavioral evidence and computational simulations in ACT-R (Dillon et al., 2013)."

Throughout the manuscript, the authors talk about different content-addressable architectures with a cue-based retrieval mechanism, namely, McElrees conception of a content-addressable memory on the one hand side and ACT-R on the other hand. However,

these two accounts differ in important aspects that are crucial for the author's research question: both architectures assume that similarity-based interference affects the accessibility of an item in memory. The authors translate the concept of accessibility to an items activation in ACT-R (page 14) (which I fully agree with). However, whereas the probably most important claim of McElree's account is that retrieval times should NOT be affected by similarity-based interference, in ACT-R, the retrieval latency of an item is actually a function of its activation, which in turn is a function of spreading activation that depends on similarity-based interference. The authors should clearly spell out the differences between the different cue-based retrieval models and specify the predictions of which one they are testing in their experiments. Moreover, they should mention that not all studies showing interference are indeed evidence for McElree's account, but are rather uninformative as they do not use paradigms that allow to tease apart accessibility and retrieval latency (according to McElree methods such as SPR or Eyetracking are not suitable to test whether retrieval times are constant).

We thank the reviewer for drawing our attention to the fact that our study does not allow to take a position with respect to the specific architecture of the content-addressable memory system assumed to underlie the effects reported. We now mention ACT-R as a potential one, and reduced reference to it.

Relatedly: page 11: "... evidence shows that semantic and syntactic similarity affect the accessibility of the element to be retrieved, but not the speed with which it is retrieved. This, again, supports the hypothesis that constituents are retrieved on the basis of their content, by way of a cue-based retrieval mechanism." Clarify that not all cue-based retrieval make this predictions. The ACT-R cue-based retrieval model predicts that retrieval latencies are indeed affected by semantic and syntactic similarity.

This is now clarified in the revised ms.

Predictions: Related to the above point about coding of the attraction effect, I am not fully convinced that the authors correctly derive the predictions of McElree's cue-based retrieval account: For experiment 1, the authors claim, that cue-based retrieval (in McElree's view, I assume) predicts "(a) generally more errors and/or slower response times in the presence of a mismatching plural attractor". First, I don't understand why the authors predict slower response times --- their hypothesis is that access time is constant, i.e., not affected by similiarity-based interference.

With respect to this first point, indeed, the focus on content-addressable memory models in the introduction let the reader think that our predictions directly derived from them. However, our working hypothesis, as stated in the Overview of the study, is more limited in stating that “the memory access for elements from the sentence operates on hierarchical representations and that agreement/attraction is tightly linked to the properties of items in memory”. The predictions are thus restricted to the alignment between the degree of interference observed in Experiment 1 and the memory parameters expected in Experiment 2 (dynamics and accessibility). To avoid confusion, we suppressed the third prediction, which was more directly linked to properties of content-addressable models, and refer the reader to the General discussion for a discussion of our results in regard to those models (we agree

that content-addressable models do not predict that the strength of interference should vary with the dynamics of the interfering element but with its accessibility). The section of the General discussion entitled “Hierarchical memory architecture underlying attraction” which reviews evidence that attraction is an instance of similarity-based interference now starts by highlighting our observations from Experiment 2, also in support of content-addressability:

“In the introduction, we reviewed evidence that memory retrieval, for lists and for sentences, is content-addressable. Our findings from Experiment 2 that the strength of attraction coincides with the level of accessibility of the attractor, and not with its retrieval speed, is in line with the hypothesis that the influence exerted by an attractor on sentence processing lies in a content-addressable mechanism relying on cues, rather than on a mechanism relying on search (McElree, 2006). Additional evidence for content-addressability would come from the observation that attraction is sensitive to similarity. (…)”

Second, I don't understand why in the grammatical conditions (i.e., conditions with a singular target and a singular verb), a plural attractor should induce interference. I think that, in a content-addressable framework, a singular attractor should induce interference, i.e., misretrievals in McElree's account.

With respect to this second point: multiple reports from the literature as well as data from Experiment 1 show that when participants are asked to perform a grammaticality judgment task, grammatical sentences with a plural attractor induce interference. We do not think it is incompatible with content-addressable models since, as we discuss it in the Discussion of Experiment 1, grammaticality judgment taps into a different process from passive parsing: we suggested that it taps into the process of agreement checking for which agreement features cannot act as retrieval cues (since they need to be checked), such that the misidentification of the attractor as being the agreement controller can only lead to a detectable error when the attractor mismatches the controller, exactly like what is found in sentence production.

page 15ff: Introduction of SAT:
- the greek letters are not displayed in the main text, which makes it impossible to review the authors' claims.

We are unsure why this happened, and we regret the inconvenience it caused. We have attempted to replace all symbols to map to Times New Roman, and the letters seem to be correctly displayed in the current version of the PDF.

- page 15, d' equation: The case distinction is not mathematically sound: The cases are not mutually exclusive; a posssible fix is to write t>delta>0 for the first case and "otherwise" for the second;
- page 26: the d' equation is different from the one presented on page 15.
- page 15: the statement for t=1/(1/beta) is incorrect. Do the authors mean t=1/beta?

We have corrected these inconsistencies in the presentation, and use the same equation, with the same typesetting in both presentations. The case distinction is now corrected.

- page 19: "Participants gave no response on approximately 11% of trials and these trials were removed from further analysis.": Why didn't participants respond? Because of a time-out? How much time did they have to respond? Given the extremely small sample size for 16 conditions, a data loss of 11% is considerable.

We now incorporated more information about the procedure, including the fact that there was a 5000 ms timeout. While this cannot address the reviewer's broader concern about data loss – since we cannot analyze the trials on which no response is given – it does give context to the 11% figure.

Analysis of Experiment 1:
I did not understand, why the authors constructed "Attraction Index" as a dependent variable (page19ff). First, there is the above mentioned issue about the coding of the "attraction" effect in grammatical conditions. Second I do not understand why the authors compute an Attraction index by subtracting the accuracy scores in the match and mismatch-conditions and then use this Attraction Index as dependent variable in a linear mixed model with match/mismatch as a predictor. Why don't the authors simply code match/mismatch as a fixed effect in a (hierarchical) logistic regression? This would be much more straightforward and, additionally, line up with the analysis of the RTs.

We did this in an attempt to construct an analysis in a way (that we thought) would be more understandable to the reader; and to have an index to compare with the SAT parameters.

Practically a hierarchical logistic regression is problematic for the judgment data because there are many conditions, participants and items without any errors. We included an ordinary logistic regression model using Firth's penalized likelihood method to address the issue of quasi-complete separation that we observed.

Selection of the predictors: According to the model description on page 19, the authors did not include any interactions in their models, why? In particular, the interaction between grammaticality and match is theoretically very relevant.
- page 21: three-way interaction: the authors did not mention in the model specification that they were also testing interactions. Please do so.

We had included interactions. This has been clarified in the revised ms.

Analysis of RTs: why did the authors remove incorrect trials? How much data was excluded? This was in addition to the 11% of removed data due to missing responses, correct? Please be more explicit about the total amount of excluded data.

Here we follow the standard practice of separating correct and error trials for analyzing RTs. There just happen to be relatively few trials, but this can be seen explicitly with the inclusion of Table 2.

- For all analyses provide the effect estimates, not only t- or p-values.

Done.

- page 21: in order to interpret a three-way interaction, the authors "computed an RT Attraction index for each tuple by subtracting RTs in the Match condition from RTs in the Mismatch condition, thus showing how much a plural attractor disrupted judgment." I don't understand why the authors not simply successively resolved the interaction by fitting models with pairwise comparisons (i.e., the two relevant two-way interactions nested within the third factor).

We were attempting to match the attraction index analysis before to explicitly show the cost that a mismatching attractor has on (correctly) judging a sentence. We have replaced that analysis with a pairwise comparison, as the reviewer suggests.

Analysis Exp 2:
"We fit a fully-saturated model to each participants' data" How? Maximum likelihood? Please be explicit about this.

Model fitting detail is now given on p. 28.

"While it is conceivable to separately analyze beta and delta parameters, practically they trade off during the estimation process." Why and how? please clarify.

We have inserted some greater explanatory text. In essence, it's because the rate of rise from 0 is often quite steep; and this means to get accurate, independent estimates of the intercept and the rate parameters, one would need an impracticable amount of sampling in a brief region – i.e., many more response signals. In contrast, information about the asymptote is carried in many responses (from ~ 2000 ms on) and thus the estimates tend to be quite stable.

page 31: "Values were first centered around the grand mean of each experiment to allow for comparison across scales." Were they also normalized (z-score, min-max...?) Centering alone doesn't make scales comparable.

This was an unintentional omission. The values were normalized via the scale function (=z-score). More details have been inserted now.

General Discussion:
- It is misleading to start the general discussion with ACT-R since the authors did not test ACT-R predictions. As mentioned above, they should clearly distinguish the different accounts of cue-based retrieval.

We now only refer to ACT-R as one potential model among others without suggesting that our results validate it.

- "Content-addressability has the major advantage of being direct and thus fast, but the price to pay is similarity-based interference. Is attraction the consequence of similarity-based interference in sentence processing?" Here, the authors seem to imply that their coding of

attraction reflects similarity of the target and the attractor. However, this is only the case in the ungrammatical conditions, not in the grammatical conditions. See my other points above.

No, as we explained in our earlier response to that point, we found the same effect in grammatical and ungrammatical sentences, in line with previous studies based on the same experimental task.

- Also, the discussion of the literature must be revised carefully: the different studies use different contrast codings, i.e., people have referred with the term "attraction" or "interference" or "intrusion" to different experimental comparisons, or different directions of effects.

As mentioned in our earlier response, we have now added various paragraphs making this clear.

Minor issues:
Page 4: "It also accounts for the fact that elements situated higher in the subject phrase (‘flights’ in The helicopter for the flights over the canyon are low) generate more errors than those situated lower (‘canyons’ in The helicopter for the flight over the canyons are low)": These examples are not adequate here (same syntactic structure)

Indeed, the two structures are identical, what varies is the position of the plural attractor; either high or low in the subject phrase.

Page 5: "The facilitatory effect of a mismatching attractor was consistently reported in ungrammatical sentences". In the literature, the term (mis-)match sometimes refers to (mis-)match of the target and the attractor noun and sometimes to the match between the verb and the attractor. When first mentioning this term, a brief definition would be useful.

This has now been clarified:
“A number of studies using self-paced reading, eye-tracking and ERP methods have reported effects of a mismatching attractor (i.e., an attractor with a number feature that mismatches that of the subject head) taking the form of an illusion of grammaticality (…)”

page 8: "long-distant dependencies": typo?

Fixed.

page 10: "Importantly, when experimental measures allowed teasing apart accessibility and the dynamics of retrieval through the SAT methods, evidence shows that semantic and syntactic similarity affect the accessibility of the element to be retrieved, but not the speed with which it is retrieved.": Add reference.

Fixed.

page 10: "a verb that element relates to": insert article

Fixed.

*Table 1: please show *all* experimental conditions (including the "match/mismatch" manipulation)*

The match/mismatch manipulation has been added to the table.

page 18: "were split into windows corresponding to phrases": please be more specific; e.g., show regioning in Table 1.

We modified that sentence:
"Materials were presented on a computer screen using the E-Prime software. Sentences were split in windows corresponding to phrases (grammatical words were presented together with the content word they were linked to)."

Analysis: make sure all Greek characters are displayed.

We believe this is now addressed.

page 25: "Each 1000 Hz tone was 50 ms in duration and there was a lag of 350 ms between them." between onset and onset or between offset and onset?

This is now specified:
"Each 1000 Hz tone was 50 ms in duration and there was a lag of 350 ms between the offset of a tone and the onset of the following tone."

page 26ff: the authors refer to delta as intercept. I find this terminology very misleading since usually, the intercept refers to the y-axis intercept; delta, by contrast, is the null point on the x-axis. (But I believe to have seen a similar terminology in other SAT papers, so the authors should at least clarify their terminology.)

Here we follow the terminological conventions of other SAT papers in referring to delta as the intercept parameter, and we've now made it more explicit that it is the x-intercept (on p. 28).

page 31: "corresponding natural stimuli (Franck et al., 2002) Both": fullstop before "Both"

Fixed.

Reviewer #2

• In Experiments 1 and 2 there was no effect of height on attraction nor memory for the PP structures. Is a possible explanation here that in these structures, the participants realized that they only had to keep track of the subject (here it its canonical position) and either shallowly processed the following PPs by either effectively ignoring the information or leaving the grammatical structure unspecified? This strategy be more likely in jabberwocky

sentences because semantics did not need to be and would not automatically be processed.

A potential alternative explanation of the height findings in Experiments 1 & 2 for object structures relates to the idea that there is a special status of subjects in memory (pg 33). In the object structures, the "high" or c-commanding distractor appears in the first position of the sentence – normally the position a subject NP would appear in in an active sentence in French. It is clear from Exp2 that the true subject of the sentence appears to be the strongest in memory; however, the 2nd most accessible noun is the one in the high position. In the object sentences, linear primacy, grammatical height, and canonical word order preferences (i.e., subjects that typically start sentences and control agreement) are all confounded. It is unclear which is responsible for the effect. Are there structures in French that could be tested with this paradigm where these would not be confounded? This alternative explanation also relates to the point above where the NPs embedded in PPs are equally not the subject/controller of agreement of the sentence so they show similar attraction and memory effects.
Relatedly, the researchers mention the primacy issue at the end of page 27; however, I think more should be included here in terms of explanation to rule out this possibility.

It is true that in object structures, the c-commanding intervener is also in first position, which is the canonical position of the subject in French. Hence, one may argue that it is the linear property of the object in our OSV structures, rather than its structural c-commanding position, that is responsible for the higher attraction rate found in Experiment 1, and for the higher asymptote found in Experiment 2. However, we do not think that this explanation holds. First, similar object attraction was found in SOV structures as to OSV structures in French (Franck et al., 2006), both showing significantly stronger attraction than that from subject modifiers. The object is in a c-commanding position in both SOV and OSV, while the two structures differ with respect to the object's linear position. Second, c-commanding direct objects in SOV trigger more attraction than preceding indirect objects in SOV, again attesting to the special status of c-commanding attractors. Finally, SAT experiments do not demonstrate a clear primacy effect. In the systematic exploration of McElree (1996), a slight primacy effect was found on accuracy, while no effect was found on speed. Hence, although our results do not allow to exclude the possibility that primacy is responsible for the height effect found in Experiments 1 and 2, previous results suggest that it is not the case.

Some symbols did not transfer to PDF correctly (e.g., pg 13). This should be checked throughout.

We are unsure why this happened, and we regret the inconvenience it causes. We have attempted to replace all symbols to map to Times New Roman, and the letters seem to be correctly displayed in the current version of the PDF.

Reference list is not formatted in APA style.

We followed the format required by PLOS One.

Citing Gillespie & Pearlmutter, 2011 & 2013 as conflicting evidence in the description of the role of hierarchy in attraction on page 2 would be useful here to show that there are

alternative findings and explanations, which the authors report on in more detail in the general discussion.

We now refer to these papers in the introduction.

Overall, this is an interesting study and reports on findings that link attraction to memory processes. I would like to see the two major points above addressed in more detail in a revision.
