# Supplementary material for: Hierarchical structure and memory mechanisms in agreement attraction
Source: PLoS One. 2020 May 19;15(5):e0232163. doi: 10.1371/journal.pone.0232163 (PMC7237028; doi:10.1371/journal.pone.0232163)
Supplement: Attachment — Submitted filename: Response to reviewers 2.docx [file pone.0232163.s002.docx]

**Response to reviewers**

**January 2020**

Reviewers’ comments are in italics and blue.

**Reviewer #1**

*One major issue is that the authors do not give enough room to "encoding-based" accounts of agreement attraction: The authors mention the Marking and Morphing model in the introduction, but afterwards focus solely on a retrieval-based view of the phenomenon. I believe the combined findings from the grammaticality judgment study and the SAT task are equally compatible with the view that the encoding of the subject's number feature is contaminated during the encoding of the other nouns in the sentence, and that this encoding interference is sensitive to structural factors. The authors should definitely address this possibility, even if they have reasons to believe that it's not the right explanation (they dismiss Marking and Morphing as being unable to explain the grammatical asymmetry, but Hammer et al. have recently argued that it may be due to response bias).*

We agree with the reviewer that it is important to grant a role to encoding effects, given the recent literature (including our own work!), even though our results do not speak to the debate. We have now made it clear that our focus is on the hierarchical structure of memory representations. We have modified our title and our text in various places in order to delete reference to our previous underlying assumption that retrieval is the locus of attraction, and we have added two paragraphs. The first one is in the end of the section *Theories of attraction* in the introductory part, in which we explicitly take the position that both retrieval and encoding may underlie similarity effects, through different mechanisms (cue-based retrieval and self-organized sentence processing):

“Recent studies have started to cast doubt on the hypothesis that the only locus of similarity-based interference effects discussed in the sentence comprehension literature is the mechanism of memory retrieval. Indeed, memory involves not only retrieval but also encoding, and the effects reported in most of those studies can arise in either one or even both (Jäger, Benz, Roeser, Dillon & Vasishth, 2015). Teasing apart a retrieval locus from an encoding locus requires that similarity be manipulated on features that cannot be used as retrieval cues; if interference is found, this would provide evidence in favor of an encoding locus of the effect. Recent evidence suggests that indeed, similarity also affects encoding, since number similarity-based interference was found in acceptability judgments of verbal agreement in English past tense sentences, in which the verb carries no number feature, while gender similarity-based interference was found in Italian, in which the verb carries no gender feature (Villata et al., 2018). The authors argued that similarity effects arise both at encoding and at retrieval through a single mechanism of Self-Organized Sentence Processing (SOSP) responsible for building structures in memory. In SOSP, words activate treelets composed of semantic and syntactic features, which bond in virtue of their similarity. As a result, the more an attractor shares features with the subject, the more it will compete with it to occupy its position in the structure, and thus the more it will generate attraction errors (see Smith, Franck & Tabor, 2018, for a detailed description of the mechanism).”

The second paragraph is in the General discussion:

“Most of the literature on the role of memory in sentence processing has focused on the retrieval of arguments distant from their verb. However, we reviewed in the introduction evidence suggesting that memory encoding processes are also involved in agreement processing, as attested by reports showing that gender and number similarity between the controller and an attractor affects sentence processing even if those features are not represented on the verb (Jäger et al., 2015; Villata et al., 2018). The two experiments we reported here were not designed to determine whether the influence of memory on attraction arises during encoding, retrieval or both. Our finding that the memory parameter that aligns with attraction rates is attractors’ accessibility, and not the speed with which they are retrieved, is compatible with the hypothesis that stronger attractors are better encoded, as well as with the hypothesis that they are better retrieved.”

*The paper is somewhat difficult to read. The "Overview of the study" section is very long and discusses both studies, one after the other, along with the authors' predictions, which are then followed by the experiments themselves. I believe it would make more sense to reorganize the paper so that an overview of Experiment 1 is first given, followed by the description of Experiment 1, its results and their discussion, followed by the overview of Experiment 2, and so forth. As it is, by the time the reader has digested the information about SAT in the overview section, they will have forgotten the predictions for Experiment 1. It would also be nice to have a table summarizing the predictions for each of the experiments in a concise way. To increase comprehensibility, it would also be nice to be given an overview of the conditions, along with examples, for Experiment 2. I would suggest also incorporating into the examples which NP each probe type in the SAT task would correspond to.*

We are conscious that the Overview of the study contains quite a bit of information. However, we had initially written our paper without it, and realized that it was actually clearer to have the overall view. Also, this problem does not seem to have bothered the other reviewer nor the two previous ones that reviewed our earlier submission. Therefore, we thought it was more appropriate to keep it, but to end it with a summary of the predictions. Here is the paragraph we added:

“Our predictions can be summarized as follows. If the structural component of attraction is syntactic in nature and independent of the semantic content of the words and their relationships, we expect Experiment 1 to replicate the findings from natural language in our jabberwocky materials:

*Prediction 1*: Generally more errors and/or slower response times in the presence of a mismatching plural attractor;

*Prediction 2:* Stronger attraction from a plural attractor situated high and/or c-commanding the verb than from a plural attractor situated low and intervening by precedence on the agreement relation.

If our hypothesis is correct that memory access for elements from the sentence operates on hierarchical representations and that agreement/attraction is tightly linked to the properties of items in memory, then we expect the following in Experiment 2:

*Prediction 3*: Subject heads, with which agreement is usually computed correctly, should be more accessible (higher λ) and/or retrieved earlier/faster (lower δ, higher β) than the two attractors, regardless of their position in the linear word string;

*Prediction 4*: Attractors that trigger high attraction, expected to be those in a hierarchically higher position and/or occupying a c-commanding position with regard to the verb, should be more accessible (higher λ) and/or retrieved earlier/faster (lower δ, higher β) than those that trigger weaker attraction.”

As requested, we also added a table (Table 4) which illustrates the distribution of probe words in the 6 experimental conditions involving an item with the Modifier structure and a plural subject in Experiment 2.

*Like another reviewer before me, I am not convinced by the authors' use of an "Attraction Index". One reason is that it effectively removes the uncertainty involved in measuring the effect of Match by averaging over all trials for each item. Reporting the results of the logistic regression is reassuring in the sense that this uncertainty is taken into account, but I would suggest removing the "Attraction Index" from the manuscript entirely - especially as the results section of Experiment 1 reports logistic regression results before immediately switching to "Attraction Index" results, which is confusing to the reader. One page 21, "Attraction Index results on accuracy" are mentioned, which is equally confusing.*

We have switched to only using logistic regression, although we do not entirely agree here (in part because there were so many perfect performers). However, we appreciate the reviewer’s comments and the value of using a totally standardized measure. We have accordingly removed Figure 4, and described the alignment between the two experiments in prose.

*The reported results from Experiment 1 with regard to judgment RTs were confusing to me and did not seem to be discussed in any detail. On page 24, the manuscript says that "Low Object conditions showed a greater Match effect", but the reported result should refer to the Low Modifier, as the paragraph talks specifically about the Modifier conditions only. There is apparently a structural or linear effect here that is not reflected in the accuracy measures, and this fact should be discussed in the manuscript.*

We thank the reviewer for catching that error, which we’ve now corrected. We have also added a paragraph about RTs in the Discussion of the experiment:

These four key findings in Experiment 1 all rely on the accuracy measure. Response times showed a less clear distribution: they show sensitivity to feature mismatch (with slower RTs in mismatch than match conditions), which is independent of the attractor’s position in the object structure, but stronger for low than high modifiers in the modifier structure. That is, response times differ from the accuracy measure in how height affects them: for object conditions, high, mismatching attractors led to numerically longer judgment times, but the difference was not significant, although accuracy showed significantly more errors. This is unsurprising, given how variable long reaction times are. For modifier conditions, low, mismatching attractors led to significantly longer judgment times, although accuracy was not lower. Ultimately, we focus our discussion on the judgment accuracy because we believe they are more directly interpretable. As the results of Experiment 2 show, asymptotic accuracy in jabberwocky probe recognition tracks accuracy in jabberwocky grammaticality judgment. But why should participants’ RTs be longer for low, mismatching attractors in modifier conditions if they don’t lead to less accurate responses? We don’t have a clear understanding of this observation yet, but we conjecture that a string-local clash between the low, mismatching modifier attractor and the main verb could be responsible (Wagers et al., 2009, for a similar low-level plural effect). It is interesting to note that recent studies which collected both accuracy and RTs also found that whereas clear effects systematically emerge in accuracy, they either fail to emerge or are reduced in RTs (Villata et al., 2018; Villata & Franck, 2019).

*Connected to the previous point, there is a general issue of the design and interpretation of the studies being overly complex. Experiment 1 manipulates four experimental factors across two measures, and the fitted statistical models contain all the possible interactions. Combined with the low power (small sample size) of the studies, there are issues concerning both Type I and Type II error here, such that *any* effect being significant is highly likely given the large number of comparisons, and that an effect *not* being significant does not signal the absence of an underlying true difference between conditions. The authors should explicitly acknowledge these issues in the paper.*

Although we appreciate the issues raised by the reviewer here, we respectfully decline to make these blanket assertions that are true of any comparable study (e.g., of an effect not being significant not indicating no true difference). If there is a concern over a specific finding, we would address it. The full details of the design and analysis are given as explicitly as possible, and the underlying data and analyses will be published with the paper.

*I do not understand the point made on page 26 about the difference between reading for comprehension and grammaticality judgments. Specifically, for grammaticality judgments, I fail to see how "the agreement features of the verb, which need to be checked, cannot be used as retrieval cues". To me, it is perfectly sensible to assume that the verb triggers a retrieval for an agreement-licensing subject in these cases.*

We have reformulated our explanation, to make it clearer:

“Finally, attraction was found for both grammatical and ungrammatical sentences, and manifested as penalty due to number mismatch. Although this finding aligns with other studies that used a grammaticality judgment procedure in German (Haüssler & Bader, 2009) and in French (Franck et al., 2015, Experiment 3), it contrasts with findings from comprehension studies based on reading time tasks in which the presence of a mismatching feature was found to facilitate the reading of ungrammatical sentences (Dillon et al., 2013; Tanner et al., 2014; Wagers et al., 2009, see Jäger, Engelmann & Vasishth, 2017, for a meta-analysis) as well as grammatical ones (Franck et al., 2015; Villata et al., 2018; Villata & Franck, 2019). In line with Franck et al. (2015), we suggest that the difference comes from how the two tasks may differentially encourage use of distinct cues or distinct mechanisms. In reading, the verb is presented with its agreement feature, such that this feature can be used to retrieve the subject and build the sentence’ structure, a process which is facilitated if the attractor carries a different feature, due to the lower feature overlap between the verb and the attractor. In grammaticality judgment, although the verb carries an agreement feature, this feature is erroneous in a certain proportion of the trials and the task requires the feature be checked. Therefore participants may attempt to check the feature by covertly producing the verb phrase based on the subject. Crucially this would also affect grammatical sentences. And as a consequence, misidentification of the attractor as the agreement controller can only lead to a detectable error when the attractor mismatches the controller, exactly like what is found in sentence production.”

*Also, in their response to Reviewer #1, the authors say that "the misidentification of the attractor as being the agreement controller can only lead to a detectable error when the attractor mismatches the controller" - while this is true, it appears to me that it can be seen as a flaw of the task, given that there may be errors that are not identifiable as such (wrong agreement controller with correct feature). I believe this point should be acknowledged.*

We have now acknowledged that point more explicitly:

“It is important to note that in sentence production, the cue-based retrieval mechanism cannot rely on agreement cues on the verb to retrieve the subject, since no such feature is present on the verb, such that other cues are assumed to be used for subject retrieval (being a NP, carrying nominative case, occupying a particular phrasal or linear position). Hence, whereas in sentence comprehension similarity-based interference can manifest in terms of penalty due to similarity in agreement features of the subject and the attractor, no such penalty can manifest in sentence production. In fact, any subject retrieval error in production studies can only show up in sentences involving a feature mismatch between the subject and the attractor, such that the effect of agreement feature similarity in production can actually simply not be studied with classical production tasks, even though much of the production literature has capitalized on that effect (Schmid, Villata, Tabor & Franck, 2018). Nevertheless, similarity in terms of other features (semantic, syntactic, morphological) can be studied, and has been argued to negatively affect production (Franck, 2017, see General discussion).”

*The authors argue that the results of Experiment 1 and Experiment 2 align, and that probe recognition performance maps onto agreement attraction strength. While this interpretation is generally sensible in light of the results, retrieval in sentence comprehension appears to be more akin to a recall than a recognition task. I would like the authors to comment on this, as well as on the fact that recognition dues not necessarily involve matching of any grammatical cues.*

We now acknowledge this point when we present the probe detection task in the Overview of the study section (around p. 16):

“One may object that the probe recognition task could be performed by a general assessment of the probe’s familiarity, without the recovering of specific source information that is assumed to take place in retrieval in sentence processing. That is possible, but as familiarity is itself a way of talking about the strength of the probe’s representation (i.e., its activation), the results will nonetheless be relevant. If our participants could perform this task without recovering source information, then we may lose information about the dynamics of retrieval from different syntactic positions. However, other SAT studies testing the relative combination of familiarity and source information find that probe recognition is usually mediated by a combination of both (Hintzman & Curran, 1994, McElree, Dolan & Jacoby, 1999). Moreover, we don’t think our participants could respond accurately without recovering source information: in our study design the same probe occurred on multiple trials, either as the positive or negative probe – so sometimes a familiar probe would have to be rejected, if it were not in the most recently processed sentences.”

*Furthermore, it is somewhat troubling to me that the results of the grammaticality judgment task of Experiment 2 were not analyzed - can the authors justify this decision, given that it would be good to see a replication of the results from Experiment 1?*

We were trying to manage complexity, and did not intend to imply anything was amiss. Since we omitted the Accuracy Index analysis, we now include a (brief) analysis of these data around pp. 34-35 (Table 6). They show overall high levels of accuracy, higher for grammatical sentences, and a dependency between probe recognition and judgment: when asked to judge a probe word that turned out to be subject, accuracy was higher:

“Finally, we turn to participants’ performance in the secondary grammaticality judgment task (made after each SAT probe recognition judgment). While we had no specific predictions for this task, the results showed that participants could discriminate grammatical from ungrammatical jabberwocky sentences. They attained accuracy, on average, of 80% for grammatical sentences (the critical SAT trials) and 71% for ungrammatical sentences (the SAT fillers). Table 6 further breaks down accuracy on the grammatical trials by Structure, Probe status (Target, Distractor), and Probe (Subject, High, Low). We computed a mixed-effects logistic regression of grammaticality judgment accuracy on the factors Structure, Probe & Probe status (with the same contrast coding as above; Probe status was sum coded, with Present: +1/2). In accuracy, we found higher performance for Object than Modifier conditions (β_0_: 1.78; β_Structure_: 0.46, z=9.9, p<.001), for Subject than Non-subject conditions (β_PrbWdSubj_: 0.34, z=6.8, p<.001), and for Targets than Distractor conditions (β_PrbType_: 0.28, z=6.0, p<.001). These effects were qualified by 2 interactions: (i) Structure and Probe Word Height (β_Strct⨉PrbWdHght_: -0.24, z=-2.1, p=.035), reflecting slightly better accuracy (~2%) in High compared to Low conditions for Object conditions, but vice versa for Movement conditions; and (ii) an interaction between Probe status and Probe (β_PrbType⨉PrbWdSubj_: 0.40, z=3.9, p < .001), reflecting the fact that Probe status had its strongest effect when the probe word was the Subject (86% present v. 81% absent).

Although we are hesitant to read too much into performance in this task, given how demanding the SAT phase was, it is notable that participants could nonetheless discriminate grammatical jabberwocky sentences from ungrammatical ones. And, crucially, when they were probed for the subject head noun in the probe recognition, their performance improved on grammaticality judgment. This gives us some added confidence that the same syntactic representation was used to complete the two tasks.”

*A previous reviewer took issue with the fact that predictions from a retrieval model based on ACT-R (Lewis & Vasishth, 2005) were conflated with those of non-ACT-R models. The authors apparently responded to this criticism by removing references to ACT-R from the manuscript. Like the previous reviewer, I would like the authors to instead discuss the predictions of the different models with regard to processing speed in the SAT task, as the ACT-R model predicts that speed *should* be affected by the agreement attraction manipulation.*

We inserted a discussion of this point on pp. 18-19. It’s true that ACT-R makes a prediction about the time it takes to instate a chunk in the retrieval buffer via its latency equation, but it is possible to generate no differences in retrieval dynamics in SAT since there are other relevant parameters (Schneider & Anderson, 2012). Here is the paragraph we inserted:

“Predictions 3 and 4 are agnostic about whether effects of prominence in memory will be reflected in accessibility or retrieval speed. We adopt this agnosticism for two reasons. Firstly, the bi-partite model of working memory advocated by McElree (2006), if taken at face value, suggests that changes in the activation of particular encodings should only affect the ultimate likelihood of retrieval, and not retrieval speed. The retrieval speed, in this view, is affected primarily by whether or not the required information is in the focus of attention, or not. However, we understand so little at present about how linguistic information flows through the focus of attention, that *a priori* it is conceivable that some hierarchical effects in memory could be accounted for by how the focus of attention is managed. Secondly, ACT-R, which has been adapted to explain sentence processing (Lewis & Vasishth, 2005), explicitly links activation with latency to retrieve a chunk, with higher activations mapped to shorter latencies. How that linking hypothesis translates into SAT data is complicated, because there are more parameters than just the retrieval latency equation in a full ACT-R model; depending on time-to-encode and response strategies, differences in activation can nonetheless be manifested in asymptotic differences only, with very small effects on dynamics (Schneider & Anderson, 2012).”

*In their response to Reviewer #2, the authors explain why they do not think that the claimed effect of c-command reduces to an effect of primacy. Yet, in the manuscript proper, it is simply stated on page 32 that "we do not consider the explanation very likely given the strength of the effect". I think the full explanation should be given in the manuscript.*

We now included a more complete justification of our position:

“One may argue that the higher accessibility of the c-commanding attractor as compared to the lower one is due to its position at the beginning of the sentence. Such a possibility is unlikely, for two reasons: the primacy advantage for the first position is known to only weakly affect accessibility in SAT experiments (McElree & Dosher, 1989; McElree, 1996; McElree 2006), and, in line with those previous reports, our own findings show that the accessibility of subjects situated in first position in the sentence (in the Modifier condition) does not differ from that of subjects situated further in the sentence, i.e., before the verb (in the Object condition).”

*Minor points*

*On page 8, "The asymptote provides a measure of the overall probability of correct retrieval" should be changed to "maximum probability" if I understand the reasoning behind SAT correctly.*

We changed it to “ultimate”; we understand the potential confusion here, but also “maximum” did not seem accurate either.

*Also on page 8, "the asymptote decreases gradually with recency of list position" - I believe the authors mean "increases", looking at McErlee & Dosher (1989). The authors make the same claim again on page 39 ("accessibility is typically found to increase with linear distance to the probe"), so maybe I am misunderstanding something.*

We have changed our wording to make this clearer (the confusion stems from the fact that list position is sometimes talked about from the end, and sometimes from the beginning – it decreases as one moves further back from the probe).

*On page 11, Lewis & Vasishth (2005) should be cited as a model that assumes a narrow focus of attention (a single-element buffer).*

We inserted this reference on p. 9 (it seemed less relevant to us on p. 11).

*On page 20, the authors state that 500 ms per region were chosen "to minimize judgment errors". Are agreement attraction errors not judgment errors?*

That sentence was indeed unclear. We changed it into:

“These rather long presentation windows were chosen given the unfamiliarity of the jabberwocky words.”

*On page 28, I would like to know more about how the sessions were conducted. Did they all take place on one day or were they spread across different days? Especially if they were spread across several days, participants may have developed strategies.*

Sessions were spread over a week, as is common practice for SAT experiments (it is impossible to run them over a single day). It is unclear what kind of strategies participants may develop, and how the spreading of sessions over a day vs. several days would change those strategies (that is, strategies are possible in both scenarios).

*Typo on page 29, "that it *refers* specifically to the x-intercept".*

Done.

*On page 31, I think the authors mean "a significant interaction between Probe type and Structure" rather than "a significant interaction between Subject probes and Structure".*

Correct – thank you.

*On page 33, I am not sure if something can "be agreed with" something else in standard English.*

We adjusted (though this formulation has been validated by native English speakers!).

*On page 34, I am not convinced that the Jabberwocky sentences "ensured that the task taps into structural processes" - all they did was ensure that people did not resort to semantics for additional guidance, I think. See the point about c-command being confounded with primacy above.*

We changed the sentence into:

“we used jabberwocky materials to reduce semantic influences and maximize the role of structural information in performing the task, (…)”

*On page 36, "the presence of an attractor matching with the verb gives rise to the erroneous retrieval of that element, satisfying the parser" - add "sometimes".*

Done.

*On page 36, regarding absence of attraction effects in the presence of case marking: Recent work by Avetisyan et al. (https://osf.io/tpyjm/) suggests that case marking reduces attraction in production, but not necessarily in comprehension.*

Thank you for this reference. We now refer to it in the section on similarity effects in attraction in comprehension.

**Reviewer #2**

*Apart from a number of line-by-line comments (see further below), there are three more general points for revision that I would like the authors to consider:*

*(1) Reporting of p-values:*

*Reporting p-values as “p < .05”, “n.s.” etc. appears rather old-school and is neither very informative nor helpful for potential meta-analyses (p-curve etc.). Throughout the results sections, I would prefer if actual p-values (rounded to 3 decimals) were being reported, both for significant and non-significant effects. The only exception is “p < .001” (i.e., when the actual p-value is smaller than .001).*

This is a very reasonable concern, and we have adjusted the manuscript accordingly. Our motivation for not doing it was simply that we are publishing all the data and analyses – but it is of course more convenient to have it in the text.

*(2) Discussion of Experiment 1:*

*The discussion on p. 24-26 does not make clear whether the authors primarily rely on the ‘attraction index’ findings (Fig. 1), the RT findings (Fig. 2), or both, in drawing specific theoretical conclusions from Experiment 1. Generally, I think this section could benefit from a more thorough comparison of the results across the two DVs. For example, it is stated that “… in the double-modifier condition, both attractors generated similar rates of attraction.” (p. 25); while this appears to be true for the ‘attraction index’ data (Fig. 1), the pattern in the RT data (Fig. 2) actually looks quite different, especially for ungrammatical sentences (or am I misinterpreting something here?). Please clarify, as one could otherwise get the impression that potentially ‘inconvenient’ aspects of the results are being ignored.*

Indeed, it was not clear enough that our discussion relies on accuracy data. We have now added a paragraph in the Discussion of Experiment 1 stating this (see response to Reviewer 1), and the reason why, which is that it is generally difficult to make sense of RTs in this task, not only in our data, but also in the rest of the literature. The only studies that collected both accuracy and RTs (Villata et al., 2018; Villata & Franck, 2019) showed that whereas accuracy showed systematic effects, RTs were much less reliable.

*(3) Bounded exponential curve fitting (Experiment 2):*

*A full 12 (conditions) * 3 (lambda, beta, gamma) = 36 parameter model has been fitted to the data in Experiment 2. I appreciate that the authors are aware of the problem of parameter trade-off as a result of overfitting (cf. motivation for the 1/beta + delta measure on p. 30), but I’m not quite sure whether the measures taken to mitigate this problem are sufficient. In particular, is it safe to assume that there are no trade-offs between lambda (asymptote) and any of the speed parameters (beta, delta)? Given the large number of conditions, it would seem reasonable to determine the amount of parameter variation /necessary/ to describe the observed cross-condition differences first. This could be done via some backward model selection procedure, which would end up with a more parsimonious fixed-effects model of the data. (NB, such an approach has also been used in many of the McElree et al. papers cited).*

Because the asymptote is constrained by so many of the observations, whereas beta/delta are determined by a small number, the trade-off between lambda and beta/delta is practically much less worrisome. We appreciate this concern, and agree that backwards selection would be a potential solution. However it comes with its own problems (cf. Liu & Smith, 2009), especially given our large model space. Given that our claim ultimately comes down to variation in the lambda parameters, we propose to address the reviewer’s concern with a simpler and more direct test: one of empirical asymptotic d’. This analysis closely matches the model-fit analysis, and shows that our model-fit parameters recover the actual asymptotes (because there are only 6 scaled conditions (= d’s), it is technically an 18 parameter model). We inserted these additional paragraphs around pp. 33-34:

“Given that the major difference among conditions was in the ultimate attained accuracy, we conducted an analysis of empirical asymptotic dʹ to guard against the possibility of a fitting artefact. In this analysis, we took the dʹ series calculated for each condition and averaged the last four responses. We found empirical asymptotic dʹ matched fit dʹ quite closely. In a regression of empirical dʹ on the same experimental factors, we found a mean dʹ of 3.0; an overall effect of Subjecthood (0.27, t = 5.3, p < .001); of Structure (0.22, t=4.7, p < .001); of Height (0.12, t=2.1, p= .041); and replicated the interactions of Structure and Subjecthood (0.29; t=2.8, p = .004) and of Structure and Height (0.30, t=2.5, p = .013). These coefficients compare closely with the same coefficients from the fit dʹ model. The most considerable difference was the estimate of the Height effect: in the regression of the fit dʹ reported above, it was smaller (0.09, t=1.3, p = 0.21). On average fit dʹ values varied from empirical dʹ values less than 1%; and a linear regression of the difference between fit and empirical dʹ on the experimental coefficients showed no differences. Therefore we conclude that the differences in ultimate attained accuracy, as estimated either by fitting an SAT curve or by smoothing the last four responses, are veridical.”

*Line-by-line comments:*

We thank the reviewer for their careful reading of our manuscript, and have corrected all errors (or made changes requested).

*P. 4, second paragraph: There seems to be a noun missing in “For example, attraction was found with plural objects in the production of various involving movement of the object in preverbal position, …”*

Done.

*P. 5, second paragraph: Could you perhaps provide more example sentences here?*

Two examples are now provided.

*P. 6: What is a “clause-mate subject”? Is this an established term?*

We changed “clause-mate” to “same-clause”.

*P. 7, last paragraph: “The response-signal speed-accuracy trade-off (SAT), a procedure…”. Maybe add “paradigm” after “(SAT)”.*

Done.

*P. 12: Maybe provide some ‘double-PP’ examples here as well, not just object question examples.*

Done.

*P. 13, top: Delete “of” in “…which would attest of a direct link between attraction and memory, …”*

Done.

*P. 15: “Here, the use of the probe recognition task provides a purer measure of how well…” – perhaps replace “purer measure” with “more direct measure” (?)*

Done.

*P. 16, third bullet point after equation: The lambda parameter is not “unitless”, is it? It should be in d’ units here (i.e., indexing d’ at t = infinite).*

That is correct. We intended to said dimensionless, but that probably doesn’t help anyone. Corrected to read “in units of d’”.

*P.20, top: The description is not very transparent in terms of how the sentences were segmented for presentation. Perhaps indicate item segmentation in the examples provided in Table 1 (p. 19), using square brackets or something. NB, 500 ms per segment would hardly qualify as “Rapid” Serial Visual Presentation.*

Reference to RSVP has been dropped; we now only refer to visual presentation. Since sentences were presented word-by-word, there is no issue of how segments were defined.

*P. 21, second paragraph: What is the motivation behind analysing log RTs? Log-transforming data not only makes the data appear more ‘normal’ (less positively skewed), but also changes theoretical interpretation as it effectively turns a linear model into a multiplicative (or ‘log-linear’) one, which is not always desirable. A potentially better alternative might be to use a Gamma(identity) model family, which assumes a positively skewed data distribution without giving up the assumption of linear relationships between predictors and DV.*

This was just a typo, which we have now corrected. Our analysis was in fact in plain RTs (as the coefficients indicate), and we agree about the non-innocence of using the log transformation!

*P. 21, bottom: “ones swith” -> “ones with”*

Done.

*P. 25, end of second paragraph: “The lack of semantic information in the jabberwocky materials may have promoted more instances of the first type, …” – perhaps replace “instances” with “interpretations” (would sound better in this context).*

Done.

*P. 29, 3rd paragraph: Verb missing in “although note that it specifically to the x-intercept.” (“applies”?)*

Done.

*P. 38: “Yet, c-command differs from morphological case and semantic features in that it lies in a relational property, …” -> “… in that it constitutes a relational property, …” would sound better.*

Done.

*P. 38: “…the difference between the c-commanding object head and its modifier should have manifest…” -> better: “… should have become manifest …”*

Done.

*P. 39, under “The special status of subjects”: Verb missing in “ … independent of whether the subject is linearly to the probe …” (?)*

Done.

*P. 42, second paragraph: “This led him conclude …” -> “This led him to conclude”*

Done.

*P. 43, end of second paragraph: “structures without semantics” is perhaps a bit too strong in relation to the current materials (only the nouns were fictitious, while the verbs clearly had a meaning).*

We changed it into ‘reduced semantics’.

*P. 46: Delete “it” in “As we already discussed it, …”*

Done.
