# Supplementary material for: Hierarchical structure and memory mechanisms in agreement attraction
Source: PLoS One. 2020 May 19;15(5):e0232163. doi: 10.1371/journal.pone.0232163 (PMC7237028; doi:10.1371/journal.pone.0232163)
Supplement: Attachment — Submitted filename: Response to reviewers.docx [file pone.0232163.s003.docx]

**Response to reviewers**

**January 2020**

Reviewers’ comments are in italics and blue.

**Reviewer #1**

*While I will not insist on my general request to acknowledge the possibility of statistical errors, the authors should report confidence intervals around the model estimates throughout the manuscript (as recommended, inter alia, by the APA) to allow the reader to gauge the uncertainty of the estimates.*

- Done.

*Furthermore, the authors use a lot of space to discuss a null result (or two null results, rather - the lack of a difference between the two modifiers in both studies) in what I still suspect to be a relatively low-power situation. Here, the authors should explicitly acknowledge that we could be looking at false negatives.*

- We fully agree with the position that absence of evidence is not evidence of absence, and we now acknowledge more clearly the possibility that our negative result is due to lack of statistical power in the Discussion of Experiment 1, and postpone its discussion to the General discussion. Here is what we now say in the discussion of Experiment 1:

“Given the unexpected nature of this null result, which was not predicted by our theoretical framework, we first wanted determine whether it is paralleled in Experiment 2 before discussing possible explanations. “

- Nevertheless, we doubt that this lack of effect is due to lack of power because we found it in both experiments, and none of the two data sets showed even a trend in the expected direction, although both data sets showed a significant effect in the Object structure. Moreover, we actually did find an interaction between Match and Height in RTs in the modifier structure in Experiment 1, but in the *opposite* direction (stronger mismatch effect for the lower PP). Hence, we think that it is important to discuss this effect (making it very clear that it may still be due to lack of power), not only because it may well not be a false negative, but also because our discussion brings new ideas about the possible role of semantics and its interaction with syntactic processing, which has heuristic value for future research. Here is how we now introduce the point in the General discussion:

“Although one cannot exclude the possibility that the lack of a height effect in jabberwocky double modifiers is due to lack of power, the fact that it was systematically found across the two experiments, in contrast to the significant effect found in object modifiers, calls for explanation.”

*I also suggest shortening the section "The interplay of semantics and syntax in memory", because the differences between Jabberwocky and natural sentences may not be as important as the authors make them out to be.*

- This point is linked to the previous one, since it is in that section that we discuss the null effect in modifiers.

*In addition, I fail to see how the "Scope of planning" account of Gillespie and Pearlmutter (2011), which was proposed for production, is relevant to a comprehension study.*

- As explained in the paper, evidence suggests that the same structure-based mechanism underlies grammaticality judgements and sentence completion – probably because sentence completion involves a comprehension component. It may be clearer now, since we discuss the Scope of planning account right after having discussed the lack of a task effect. We also reframed the lack of task effect, in the following terms:

“A first difference between natural and jabberwocky data lies in the task procedure: whereas the previous findings from natural language sentences were obtained in sentence completion tasks, our current finding for jabberwocky is from a grammaticality judgment task. However, the task does not appear to be responsible for the difference: grammaticality judgments on natural French sentences were shown to give rise to stronger attraction with the highest PP (Franck & Wagers, 2015; in prep.), thus replicating results obtained with a sentence completion procedure. Independent sets of experimental evidence on other structures have shown that grammaticality judgments consistently replicate structural modulations of attraction found with the sentence completion task, suggesting that the two tap into the same mechanism of structure building (Franck et al., 2015; Haüssler & Bader, 2009, in German).”

*Detailed comments:*

*- In the abstract, "Speakers occasionally cause the verb to agree with an element that is not the subject, a so-called ‘attractor’; likewise, comprehenders occasionally fail to notice agreement errors when the attractor agrees with the verb."
I suggest a slight reformulation:
"Speakers occasionally produce verbs that agree with an element that is not the subject, a so-called ‘attractor’; likewise, comprehenders occasionally fail to notice agreement errors when the verb agrees with the attractor."*

- Done.

*- On page 4, are the authors certain that Marking & Morphing fails to predict attraction in contexts that are not the typical NP-PP-(PP) configurations? Eberhard et al. (2005:544) state that "Because SAP [singular and plural] may flow unobstructed throughout a structural network, number information bound anywhere within a structure has the potential to influence agreement processes." If I understand this statement correctly, the NP-PP configuration is not the only one where agreement attraction should occur.*

- It is true that later versions of M&M allowed any NP to influence the agreement process, which we now acknowledge:

  “Although the early formulation of Marking and Morphing assumed that only features from the subject phrase had the potential to influence the Morphing process (e.g., Bock & Cutting, 1992; Bock, Eberhard, Cutting, Meyer & Schriefers, 2001), it was later argued that any element within a structure could influence it, at a degree that depends on its structural distance from the subject (Eberhard et al., 2005).”

  However, M&M clearly fails to account for similarity effects. We now make this explicit after the discussion of similarity effects in production in the Introduction with the following sentence:

“These similarity effects are left unaccounted by the Marking and Morphing model, while they find a natural explanation under the memory-based account of attraction.”

*- On page 5, first paragraph: "it is a NP" -> "it is an NP". This pattern also appears in other places throughout the manuscript. I am not a native speaker of English, but I do not resolve the acronym to "noun phrase" when I read the text, and the form of the determiner should thus agree with the pronunciation of the acronym.*

- We changed the two instances of ‘a NP’ to ‘an NP’ (native speakers can vary considerably about prevocalic *a*, but we acknowledge the standard usage here).

*- No confidence intervals are reported for the reaction time analysis of Experiment 1, nor is there a table showing results in the same way as for the logistic regression. I would suggest adding this information so that reporting is the same across the different dependent variables. The CIs of the estimates should also be given in the text of the results section Experiment 2, so that the reader can better gauge the uncertainty of the estimates (of the differences between conditions).*

- In-line CIs are now reported throughout. We also added Table 4 to show the RT results from Experiment 1 (matching Table 3 in format).

*- There is a mismatch in the reporting of the RT results from Experiment 1 and the secondary task from Experiment 2 versus all other reported analyses in the sense that the RT/judgment results are reported with "beta(factor) = XX" whereas all other results are reported without any beta being mentioned, giving only bare numbers. The betas should appear consistently across all analyses.*

- Reporting has been made consistent across experiments. *- At the bottom of page 27, and later from page 50 onwards, the authors discuss the mismatch between their current findings and those of previous studies in terms of the absence of a difference between the two modifiers. Here, the possibility should be acknowledged that the lack of a difference in the current study may simply be a statistical false negative result, as opposed to reflecting a theoretically interesting divergence between Jabberwocky and non-Jabberwocky sentences. Looking also at the results of Experiment 2, it is of course perfectly possible that the authors are on the right track with their proposed explanation for the difference, but the more parsimonious explanation would be that the effect was masked by noise and therefore not detected in the current studies. As it is, the authors spend several pages discussing the implications of a null result, even though absence of evidence is not evidence of absence (e.g. Altman & Bland, 1995).*

- Please see our response above. *- On page 35, the authors write that "the effect of being a non-subject probe was larger in Object conditions (0.41, t=5.0, p < .001) than in Modifier conditions (0.11, t=1.9, p=.066)". But shouldn't the asymptote be higher for subjects than for non-subjects? So the estimates should either be negative or the sentence should refer to subject probes.*

- This has been re-written to make the language clearer:

“There was a significant interaction between Structure and Subjecthood (β_Strctr⨉PrbWdSubj_: 0.30, [0.15 0.44], t = 4.0, p < .001) and a significant interaction between Structure and Height (β_Strctr⨉PrbWdHeight_: 0.28, [0.10, 0.46]; t = 3.3, p < .005). To understand the source of these interactions, we created separate models on Object and Modifier conditions. In the Object condition, there was both an advantage for Subject probes (β_0_: 2.94; β_PrbWdSubject_: 0.41, [0.25, 0.56]; t = 5.0, p < .001) and an effect of Height, with High probes attaining greater sensitivity than Low probes (β_PrbWdHeight_: 0.24, [0.07, 0.40]; t = 2.5, p = .015). In Modifier conditions, there was a marginal advantage for Subject probes (β_0_: 3.15; β_PrbWdSubject_: 0.11, [-0.01, 0.23]; t = 1.9, p = .066), and no effect of Height (β_PrbWdHeight_: -0.05, [-0.16, 0.09]; t = -0.70, p = .490).”

*- On page 39, "In line with those previous reports" -> "these previous reports"*

- Done. *- Also on page 39: "The main goal of Experiment 2 was to explore the possible alignment between attraction patterns and memory. The data suggest that this is the case." The referent of "this" is unclear. I suggest changing the second sentence to "... suggest that such an alignment indeed exists".*

- Done. *- On page 51, I do not understand how the Gillespie and Pearlmutter (2011) account is relevant to the current experiments. While it is true that the Scope of planning theory takes semantic integration into account, it is fundamentally a theory of production rather than comprehension. As such, I do not see why the SOP account would predict anything at all for the current experiments, and why the authors argue that their results provide evidence against the SOP account.*

- Please see our response above.

*- On page 52, the authors are overstating their case when they say that "linear closeness to the head does not increase attraction". This statement should be changed to "Our data yielded no evidence that linear closeness to the head increases attraction". The same is true for the statement about the final sentence of the paragraph, which should be qualified be adding "in our data", at the very least.*

- Done. *- Still on page 52, the final paragraph needs to be qualified by highlighting the speculative nature of the authors' proposed explanation, and taking into account the possibility that the differences in findings are not meaningful at all but simply due to statistical error.*

- We have modified the sentence accordingly:

“Hence, assuming that the lack of a difference between the two PPs in the jabberwocky testing of double modifiers is not due to lack of statistical power, semantics had a different impact on these two structures.”

*- I fail to understand the final part of the final sentence on page 54. How do the results show that the theory of competence is also a theory of performance? I agree that performance needs to be studied to get a clear picture of what competence is, but one doesn't automatically need to equate the two. This is also the first time in the entire manuscript that the concepts of competence and performance are mentioned. Finally, I am not sure which hypothesis is being "reopened" by the findings - I'm sure that decades of psycholinguistic research were not conducted in the belief that the competence/performance debate had been settled. The authors should clarify what they mean here.*

- We agree that our point about ‘reopening’ the hypothesis was unclear, and that many psycholinguists may indeed consider that the debate has not yet been settled (but note that a number of others have considered the debate closed, discarding linguistic theory as a relevant framework to account for performance/behavioral data). It seems important to us to pinpoint that our results highlight the relevance of syntactic theory to capture performance data, and we think that the concepts of competence and performance are shared enough by the community to not need to be introduced earlier in the ms. We have modified this sentence accordingly:

“It suggests a tight link between theoretical constructs from syntactic theory, in

particular those of subject and c-command, and memory representations, and with this, suggests that the theory of linguistic competence is an important component of the theory of linguistic performance.”

**Reviewer #2**

*I am satisfied with how the authors have responded to my previous comments, which have all been addressed in the revision. I therefore endorse publication.*
